# Different Clinical Presentations and Outcomes of Disseminated Varicella in Children With Primary and Acquired Immunodeficiencies

**DOI:** 10.3389/fimmu.2020.595478

**Published:** 2020-11-05

**Authors:** Paul Bastard, Aurélien Galerne, Alain Lefevre-Utile, Coralie Briand, André Baruchel, Philippe Durand, Judith Landman-Parker, Elodie Gouache, Nathalie Boddaert, Despina Moshous, Joel Gaudelus, Robert Cohen, Georges Deschenes, Alain Fischer, Stéphane Blanche, Loïc de Pontual, Bénédicte Neven

**Affiliations:** ^1^ Service de Pédiatrie, Hôpital Jean Verdier, Bondy, AP-HP (Assistance-Publique-Hôpitaux de Paris), France; ^2^ Service d’Immunologie et Hématologie Pédiatrique, Hôpital Necker Enfants Malades, AP-HP, Paris, France; ^3^ INSERM U976—Human Systems Immunology and Inflammatory Networks, Institut de Recherche de Saint Louis, Paris, France; ^4^ Université de Paris, Paris, France; ^5^ Département d’Hématologie Pédiatrique, Hôpital Robert-Debré, AP-HP, Paris, France; ^6^ Service de Réanimation Pédiatrique, Hôpital du Kremlin-Bicêtre, Kremlin-Bicêtre, France; ^7^ Université Paris XI, AP-HP, Paris; ^8^ Université Paris Saclay, Saint-Aubin, France; ^9^ Sorbonne Université, Service de d’Hématologie Oncologie Pédiatrique, Hôpital Armand Trousseau, AP-HP, Paris, France; ^10^ Service de Radiologie Pédiatrique, Hôpital Necker Enfants Malades, AP-HP, Université de Paris, Paris, France; ^11^ INSERM U1163, Institut IMAGINE, Paris, France; ^12^ Sorbonne Paris Nord University, Bobigny, France; ^13^ ACTIV Centre Hospitalier Intercommunal de Créteil, Créteil, France; ^14^ Service de Néphrologie Pédiatrique, Hôpital Robert-Debré, AP-HP, Paris, France; ^15^ Experimental Medicine, Collège de France, Paris, France

**Keywords:** varicella, primary immumunodeficiencies, steroids, innate immunity, disseminated varicella

## Abstract

Primary infection with varicella-zoster virus (VZV) causes chickenpox, a benign and self-limited disease in healthy children. In patients with primary or acquired immunodeficiencies, primary infection can be life-threatening, due to rapid dissemination of the virus to various organs [lung, gastrointestinal tract, liver, eye, central nervous system (CNS)]. We retrospectively described and compared the clinical presentations and outcomes of disseminated varicella infection (DV) in patients with acquired (AID) (*n*= 7) and primary (PID) (*n*= 12) immunodeficiencies. Patients with AID were on immunosuppression (mostly steroids) for nephrotic syndrome, solid organ transplantation or the treatment of hemopathies, whereas those with PID had combined immunodeficiency (CID) or severe CID (SCID). The course of the disease was severe and fulminant in patients with AID, with multiple organ failure, no rash or a delayed rash, whereas patients with CID and SICD presented typical signs of chickenpox, including a rash, with dissemination to other organs, including the lungs and CNS. In the PID group, antiviral treatment was prolonged until immune reconstitution after bone marrow transplantation, which was performed in 10/12 patients. Four patients died, and three experienced neurological sequelae. SCID patients had the worst outcome. Our findings highlight substantial differences in the clinical presentation and course of DV between children with AID and PID, suggesting differences in pathophysiology. Prevention, early diagnosis and treatment are required to improve outcome.

## Introduction

Primary infection with varicella-zoster virus (VZV), a ubiquitous, human-restricted double-strand DNA alpha-herpesvirus (1, 2), causes chickenpox. In non-vaccinated populations, seroprevalence for VZV increases with age in children, reaching more than 90% by the age of 18 years in most (3, 4), but not all countries (2). VZV reactivation after a quiescent phase in the sensory ganglia (3, 4) causes shingles. The most common clinical presentation of chickenpox is a pruritic vesicular rash, beginning about 2 weeks after exposure and progressing in flares over several days, as patients typically have lesions at different stages of development (5). In previously healthy children, chickenpox is generally a mild and self-limited disease, but VZV infection in patients with primary or acquired immunodeficiencies can be life-threatening in rare cases, due to rapid dissemination of the virus, causing respiratory, gastrointestinal, hepatic, ocular (retinitis, keratitis), and central nervous system (encephalitis, meningitis, cerebellitis, central nerve palsy, vasculopathy) involvement. These disseminated varicella infections (DV) result in high morbidity and mortality (6–8). In patients with acquired immunodeficiencies (AID), diagnosis is often delayed due to unusual initial presentations (6), such as the absence of a rash, or the presence of isolated abdominal pain, with a severe fulminant course. Susceptibility to VZV infection is variable in patients with primary immune deficiencies (PID), dependent on the immune functions affected. No difference in the clinical course and outcome of disseminated VZV infection has yet been described between patients with PID and those with AID. In France, global vaccination for VZV is not currently recommended and limited to at-risk groups. We thus retrospectively analyzed 19 cases of DV in French children with acquired or primary immunodeficiencies, from 2003 to 2016. We show that these two groups have very different initial clinical presentations and outcomes, suggesting that the pathophysiology of the disease depends on the underlying cause of immunosuppression.

## Materials and Methods

### Study Design and Participants

Patients with AID were recruited via a standardized survey sent to all specialists likely to have treated children hospitalized for DV in French university hospitals over a 13-year period (from 2003 to 2016). This call for collaboration was issued via the mailing lists of the Pediatric Infectious Disease Group, the French group of intensive care units and emergency departments, the French Society for Pediatric Hematology and Immunology (SHIP), the French Society for fight against Cancer and leukemias in Children and adolescents (SFCE) and the French Society for Pediatric Nephrology. Patients with primary immunodeficiencies (PIDs) were recruited from the immuno-hematology and rheumatology unit of Necker Hospital in Paris and were identified through the hospital data warehouse (9, 10). The inclusion criteria for all patients were: i) Child or adolescent aged 0 to 18 years, ii) receiving immunosuppressive therapy or with a PID (using ESID criteria, whether or not diagnosed at the time of VZV infection) iii) hospitalized for a proven DV (at least one organ other than the skin involved; with at least one sample testing positive for VZV). Extensive varicella infections (severe mucocutaneous involvement) complicated with hemorrhagic disease only and cases of chickenpox with secondary bacterial infections only were not included. Patients undergoing hematopoietic stem cell transplantation (HSCT), patients infected with the human immunodeficiency virus and PID patients on immunosuppressive drugs were excluded. The study protocol was approved by an independent local ethics committee (*Comité Local d’Ethique pour la Recherche Clinique*; reference: CLEA-2016-029, October 12, 2016).

### Data Collection

We retrospectively reviewed the files of all patients with disseminated varicella infection and retrieved data for the patient’s personal and familial medical history, clinical and radiological features, treatment and outcome.

### Statistical Analysis

Statistics were performed using R (CRAN) version 3.6.0. We described patient characteristics as numbers and percentages for categorical variables, and median or means with interquartile ranges for quantitative ones. Wilcoxon test was used to compare quantitative variables and the Fisher's exact test for qualitative ones. Two-sided p-values < 0.05 were considered significant.

## Results

Between January 1, 2003 and January 1, 2016; 19 patients (seven with AID and 12 with PID) from six centers in France were included in the analysis. All patients had suffered from DV and satisfied the inclusion criteria. The characteristics of the patients are presented in **Tables 1**–**3**.

**Table 1:** Demographic, clinical, biological, treatment and prognosis characteristics of the 7 patients with AID included in the French retrospective study of DV in children.

Case	Age	Sex	First symptom	Clinical and biological presentation	Medical history	Immunosuppressive therapy	VZV DNA +	Delay between onset and TTT initiation (hours)	Treatment	Outcome
**N01**	16	M	Abdominal pain	Abdominal pain, seizures, skin rash, hemorrhages, hepatitis, DIC	Nephrotic syndrome, 3rd relapse	Steroids, Ciclosporin	Blood: + CSF: ND Skin: ND	38	Acyclovir IV + IgIV	Death
**N02**	14	M	Fever, vomiting	Fever, skin rash, coma, acute respiratory distress, hemorrhages, rhabdomyolysis, hepatitis	Nephrotic syndrome, 1st relapse	Steroids	Blood: + CSF: ND Skin: ND	52	Acyclovir/Gancyclovir/Foscavir IV + IgIV	Death
**N03**	12	M	Headache	Headache, convulsions, hepatitis, neutropenia, thrombopenia	Lymphoblastic lymphoma type B	Methotrexate, Purinethol	Blood: + CSF: + Skin: ND	46	Acyclovir IV, VZIG, IgIV	neurologic sequela
**N04**	6	M	Abdominal pain	Abdominal pain, skin rash, acute respiratory distress, hepatitis, renal failure, thrombopenia	Renal transplant for renal hypoplasia	Steroids, Tacrolimus	Blood: + CSF: ND Skin: ND	78	Acyclovir IV, VZIG, IgIV	Favorable
**N05**	6	M	Abdominal pain	Abdominal pain, fever, acute respiratory distress, hepatitis, CID, thrombopenia	ALL type B	Methotrexate, Purinethol	Blood: + CSF: ND Skin: ND	96	Acyclovir IV, VZIG	Favorable
**NO6**	4	F	Abdominal pain	Abdominal pain, hemorrhage, fever, hepatitis, CID, thrombopenia, skin rash	ALL pre-B	Steroids, Chemotherapy	Blood: ND CSF: ND Skin: ND	90	Acyclovir IV, IgIV	Favorable
**N07**	10	M	Skin rash	Abdominal pain, respiratory distress, fever, hepatitis, skin rash	ALL type B	Methotrexate, Purinethol	Blood: + CSF: ND Skin: +	35	Acyclovir IV	Favorable

Age is expressed in years. M, male; F, female; ALL, acute lymphoblastic leukemia; DIC, disseminated intravascular coagulopathy, ICU, intensive care unit, BAL, bronchoalveolar lavage; IV, intravenous; TTT, treatment; VZIG, immunoglobins against varicella-zoster virus; IgIV, intravenous immunoglobins.

**Table 2:** Treatments of patients with AID.

Patient	Steroid molecule	Daily steroid dosage during varicella infection	Total cumulative steroid dose	Time between steroid initiation and varicella infection	Other immunosuppressive treatments (and doses)
**N01**	Oral prednisone	2 mg/kg/day	NA	1 month	Cyclosporine (oral, 165 mg/m²/day)
**N02**	Oral prednisone	0	NA	1 month	None
**N03**	Oral prednisone	1.2 mg/kg/day	NA	2 years	Tacrolimus (oral, 0.23 mg/kg/day)
**N04**	None (stopped 5 months before)	0	1800 mg/m² (60 mg/m²/day for 1 month)	6 months	Methotrexate (oral, NS) and purinethol (oral, NS)
**N05**	None	0	NA	NA	Methotrexate (oral, 25 mg/m²/week) and purinethol (oral, 75 mg/m²/day)
**N06**	Oral prednisone (7 days, at 60 mg/m²/day) followed by IV dexamethasone (7 days)	6 mg/m²/day of IV dexamethasone	420 mg/m²/day prednisone + 400 mg/m²/day prednisone equivalent	14 days	Intrathecal methotrexate (14 days before varicella infection); IV vincristine (7 days before varicella infection); IV L-asparaginase (2 and 4 days before varicella infection)
**N07**	None (stopped 9 months before)	0	1800 mg/m² over a 6-month period	15 months	Methotrexate (oral, 25 mg/m²/week) and purinethol (oral, 75 mg/m²/day)

NA, not applicable or unknown.

**Table 3:** Demographic, clinical, biological, treatment and prognosis characteristics of the 13 patients with PID included in the French retrospective study of DV in children.

Case	Age	Sex	First symptom	Clinical and biological presentation	Medical history	VZV DNA +	Treatment	IRIS/time post HSCT/organs	Outcome
**N08**	1,5	M	Skin rash	Skin rash, stroke 6 months after the initial VZV infection	CID: MHC II deficiency	Blood	Acyclovir IV HSCT (MUD)	No	A.W, mild Neurological sequelae
**N09**	12	M	Skin rash	Bilateral interstitial pneumonia, impetiginized skin rash	CID: ARTEMIS deficiency	Blood, lungs (BAL), skin	Acyclovir IV HSCT (genoid)	No	A.W.
**N10**	0,5	M	Skin rash	Impetiginized skin rash, respiratory distress	CID: ZAP-70 deficiency	Blood, lungs (BAL)	Acyclovir IV HSCT (MUD)	Yes (M24) Encephalitis, pericarditis, skin lesions	A.W. keloid scar
**N11**	4	M	Skin rash	Severe skin rash, respiratory distress (bilateral pneumonia)	CID: ZAP 70 deficiency	Skin	Acyclovir IV No HSCT	No	A.W.
**N12**	3	F	Skin rash	Severe skin rash, bilateral VZV pneumonia	CID (unidentified)	Blood, lungs (BAL)	Acyclovir IV No HSCT	No	A.W. Keloid scar
**N13**	0,8	F	Skin rash	Respiratory distress, skin rash	CID (unidentified)	Blood, lungs (BAL)	Acyclovir IV HSCT (MMRD)	No	A.W.
**N14**	1,3	M	Skin rash	Skin rash, ARDS, keratitis, meningo-encephalitis	SCID: JAK 3 deficiency	Blood, lungs (BLA), skin, CSF, vitreous fluid	Acyclovir IV + Foscavir IV intra-vitreous Foscavir HSCT (MMRD)	Yes (M3) pneumonitis, retinitis	Death from suspected IRIS
**N15**	1	M	Skin rash	Severe skin rash, bilateral interstitial pneumonia	SCID : RAG 2	Blood	Acyclovir IV + Foscavir IV HSCT (MMRD)	Yes (M2) encephalitis	Death from suspected IRIS
**N16**	0,7	F	Skin rash	Skin rash, bilateral interstitial pneumonia, radiculo-neuritis	SCID: RIL-7alpha	Blood, CSF	Acyclovir IV + Foscavir IV 2 HSCT (MMRD)	Yes (M4) Pneumonitis	Death from suspected IRIS
**N17**	0,2	M	Skin rash	Severe skin rash, meningo-encephalitis	SCID : RAG 1	Blood, CSF	Acyclovir IV HSCT (genoid)	Yes (M1) Encephalitis	A.W., mild Neurological sequelae
**N18**	0,4	F	Meningitis	Respiratory distress, neurological involvement (meningitis, retinitis)	SCID: γC	Blood, CSF	Acyclovir IV + Gancyclovir IV followed by Foscavir and Cidofovir. HSCT (MMRD)	No	Severe Neurological and ocular sequelae
**N19**	0,5	M	Skin rash	Severe skin rash, neurological involvement, hepatitis	SCID: RAG 1	Blood, CSF	Acyclovir IV HSCT (MMRD)	No	Death from VZV encephalitis 2M after HSCT

Age is expressed in years. M, male; F, female; ALL, acute lymphoblastic leukemia; SCID, severe combined immunodeficiency; CID, combined immunodeficiency; HSCT, hematopoietic stem cell transplantation; RAG, recombination-activating genes; ARTEMIS, Artemis protein, encoded by the DCLRE1C gene; ZAP, 70 kb zeta chain-associated protein kinase; BAL, bronchoalveolar lavage; IV, intravenous; TTT, treatment (500 mg acyclovir/m^2^/8 h).

### Disseminated Varicella Infection in Seven Patients With Acquired Immunodeficiencies

In patients with AID (**Table 1**), the mean age at DV diagnosis was 10 years (range: 4–16 years). All but one of the patients were male. The underlying conditions were steroid-dependent nephrotic syndrome (*n*=2), renal transplantation (*n*=1) and malignant hemopathies (*n*=4). Immunosuppressive treatments included corticosteroids, cyclophosphamide, tacrolimus and/or a combination of methotrexate and purinethol. The durations and doses of the immunosuppressive treatments are shown in **Table 2**. Four of the seven patients were on corticosteroids at DV onset, and all patients had received corticosteroids as part of their treatment during the preceding 6 months. The median duration of immunosuppressive therapy before DV was 36 months (range: 0.5–168 months). All patients were living in France and none had been vaccinated against VZV. The index case of varicella infection was identified for only one of these patients, who then received prophylactic acyclovir.

### Symptoms of Disseminated Varicella Infection in Patients With Acquired Immunodeficiency

The first symptom was abdominal pain in four of the seven patients, and all patients presented abdominal pain during the course of infection; five had bilateral hypoxic varicella pneumonia requiring oxygen therapy, and two presented seizures (one with confirmed VZV encephalitis). Five of the seven patients had a skin rash, but with an onset at least 48 h after the first symptom in all but one case, this last patient presenting a rash at onset. High liver enzyme levels were noted in all cases, and four patients developed fulminant hepatitis with acute liver failure. High pancreatic enzyme levels were also recorded in four cases, but there were no cases of severe pancreatitis. Two children had disseminated intravascular coagulopathy, including one with severe hemorrhagic syndrome. Two instances of concomitant infections were noted (*Candida albicans* septicemia and *Pseudomonas aeruginosa* septicemia, in one patient each). All patients were admitted to an intensive care unit (ICU), for a median of 11 days (range: 2–27 days). The median time between disease onset and treatment initiation was 52 h (range: 35–96 h). All patients received intravenous acyclovir for 14 to 21 days (500 mg/m^2^/8 h in all cases). Six patients also received immunoglobulins: specific anti-VZV immunoglobulins (*n*=1), polyvalent immunoglobulins (*n*=3), or both (*n*=2). Immunosuppressive drugs were suspended in all cases. Two patients died from multiple organ failure, nine and 30 days after disease onset. Outcome was favorable in four patients, who made a full recovery, and one patient survived but experienced neurological sequelae.

### Disseminated Varicella Infection in 12 Patients With A Primary Immunodeficiency

In patients with a PID (**Table 3**), median age at DV onset was 0.9 years (range: 0.2–12). Four of the patients were female and eight were male. In all patients, DV led to the diagnosis of the PID. Six patients suffered from severe combined immune deficiency (SCID) (γC deficiency *n*= 1, IL-7 receptor deficiency *n*= 1, JAK 3 deficiency *n*= 1, RAG-1 deficiency *n*= 2 and RAG-2 deficiency *n*= 1), and six had a combined immune deficiency (MHC class II deficiency *n*= 1, ZAP-70 deficiency *n*= 2, hypomorphic ARTEMIS deficiency *n*= 1, and combined immune deficiency with an unknown molecular diagnosis *n*= 2). None of the 12 patients or their relatives had been vaccinated against VZV.

### Symptoms of Disseminated Varicella Infection in Patients With Primary Immunodeficiency

**Figure 1:**
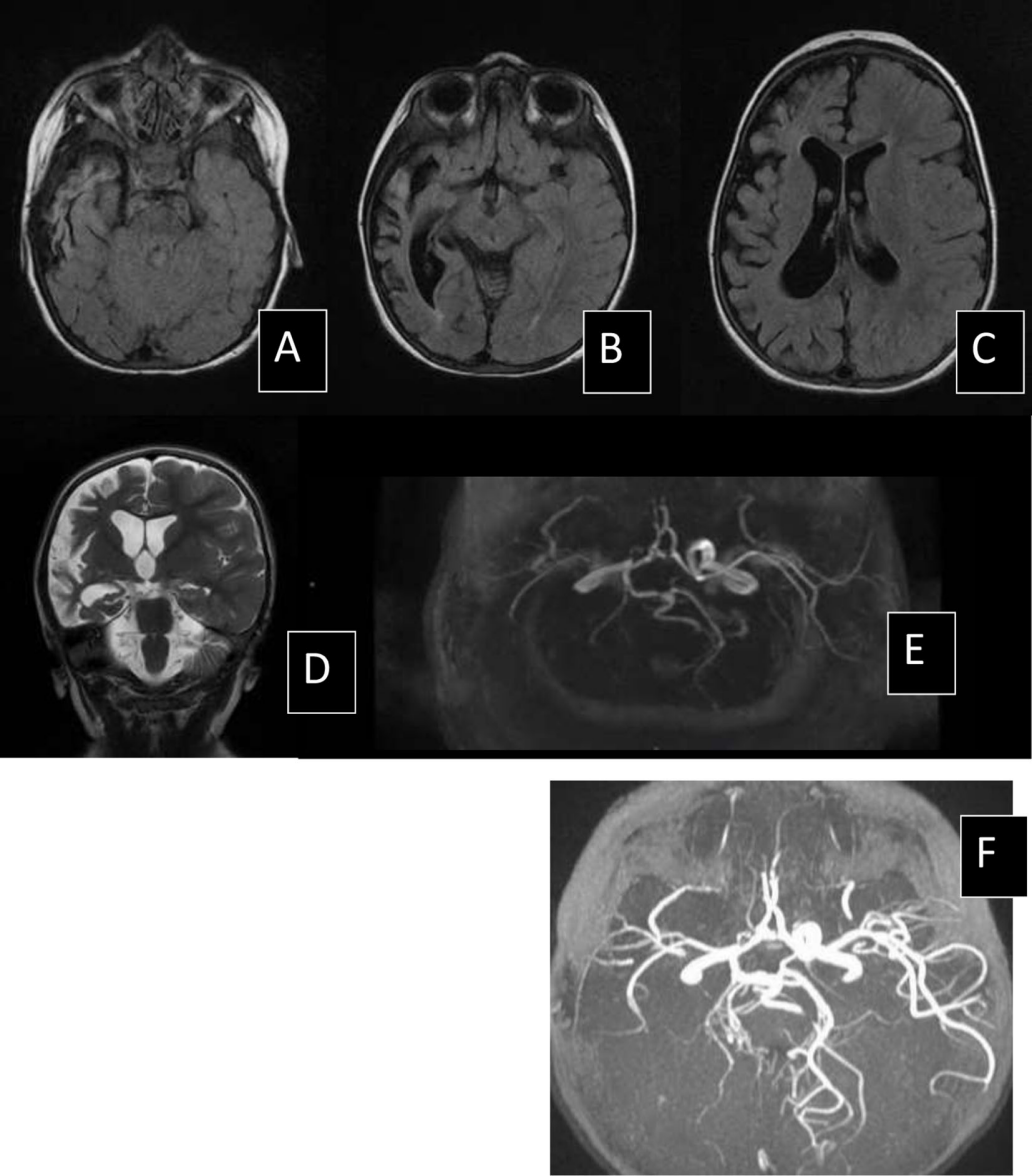
MRI scan of patient N09, 1 year after stroke secondary to cerebral VZV vasculitis. Right hemisphere atrophy on axial FLAIR sequence **(A–C)** and coronal T2 sequence **(D)** and stenosis of internal carotid artery and posterior cerebral arteries in vascular sequences Time of flight (TOF) angiography **(E, F)**.

The first symptom was disseminated skin rash in all but one of the patients. Other manifestations included neurological symptoms (*n*= 6) (meningo-encephalitis in five patients and radiculo-neuritis in one case), VZV-retinitis (aqueous humor positive for VZV) in one patient and pneumonitis in nine patients (with a positive PCR test for VZV on bronchoalveolar lavage for all five patients tested). One patient had a stroke following VZV vasculitis (**Figure 1**). Two patients had high liver enzyme levels, but none presented acute liver failure. Three patients were admitted to the ICU at onset, due to respiratory distress. The symptoms of varicella infection were remarkably prolonged and required the administration of more than one intravenous antiviral drug, concomitantly and/or successively, in five cases. Treatment was prolonged in all patients and was maintained until immune recovery after hematopoietic stem-cell transplantation (HSCT) in 10 patients, or until complete clinical recovery and an undetectable viral load for VZV in the two CID patients who did not undergo HSCT. All patients received polyclonal immunoglobulins, but none received VZV-specific immunoglobulins. All four of the patients who died had SCID. Death occurred three to 4 months after HSCT, due to suspected immune reconstitution inflammatory disease (IRIS) (*n*= 3) or disseminated VZV infection (*n*= 1). Five of the eight patients who survived suffered from severe neurological sequelae (*n*= 3) and/or skin sequelae (diffuse keloid scars *n*= 2). As expected, the course of the disease was more severe in patients with SCID, four of whom died, with two others surviving but experiencing neurological sequelae. All six patients with CID survived. One patient had neurological sequelae and two had keloid scars, whereas the other three of these patients had no sequelae. Of note, ten PID patients underwent HSCT, and IRIS was suspected in five of these patients after HCST.

### Differences in the Clinical Characteristics and Outcomes of Disseminated Varicella

Significant differences in the clinical characteristics and outcomes of DV were observed between patients with AID and PID, as highlighted in **Table 4**. First, all the patients with AID were older than patients with PID (p=0.002, Wilcoxon test). Furthermore, patient with AID had a severe, fulminant clinical presentation, requiring hospitalization in the ICU for severe symptoms, such as multiple organ failure and coagulopathy. PID patients had an acute but not fulminant presentation, with persistent infection. At onset, the manifestations of the disease differed significantly between the two groups. Abdominal pain and hepatitis (*p*<0.0001 and *p*<0.001, respectively) were more frequent, and skin rash was delayed or absent with AID relative to patients with PID. Mortality was high in both groups, but sequelae were more frequent in PID patients, due to the infection itself, or possibly due to IRIS occurring after HSCT.

**Table 4:** Comparison of clinical characteristics between patients with DV and AID or PID.

	Acquired ID	Primary ID	Statistical analysis*	Test used
**Age (mean)**	9,2	0.9	0.002	#
**Sex (M/F)**	6/1	8/4	0.6	*
**Clinical and biological manifestations:**				
** Abdominal**	7/7	0/12	*p* < 0.0001	*
** Neurological**	3/7	6/12	NS	*
** Cutaneous**	3/7	12/12	*p* < 0.01	*
** Hepatitis**	7/7	1/12	*p* < 0.001	*
**Treatment**				
** >1 antiviral drug**	6/7	4/12	*p* = 0.057	*
**Severity:**				
** ICU**	7/7	3/12	*p =* 0.003	*
**Outcome:**				
** Sequelae**	0/5	5/8	*p* = 0.075	*
** Death**	2/7	4/12	NS	*

Age, expressed in years. M, male; F, female; DIC, disseminated intravascular coagulopathy; ICU, intensive care unit. ^#^Wilcoxon rank sum test with continuity correction. *Fisher's Exact Test for Count Data.

## Discussion

This study highlights substantial differences in the clinical presentations and outcomes of DV between patients with AID (due to corticosteroids and/or other immunosuppressive drugs) and patients with PID (i.e. T-cell deficiency in this series). Indeed, we observed a significantly older age in patients with AID. The disease also followed a more fulminant course in AID patients, with an early abdominal pain but a delayed rash as previously described (8, 11, 12), whereas patients with T-cell deficiency presented a more typical rash, with wide dissemination, persistent infection, a higher rate of sequelae and IRIS-related complications during immune reconstitution after HSCT. Mortality was high in both groups. It is not possible to draw definitive epidemiological conclusions from this study due to its retrospective nature and the potential underreporting or underdiagnosis of cases, particularly in AID patients.

These significant differences may be due to the underlying defect of immunity. Two distinct viremic phases occur after the natural acquisition of VZV. The initial phase is asymptomatic in immunocompetent hosts, occurs 5–7 days after inoculation, and engages innate immune responses, especially type I interferon production. The second phase of viremia begins after 11–21 days (5), when the skin rash occurs, and corresponds to the onset of specific adaptive immune responses. Early innate responses are important for triggering and amplifying the adaptive immune response leading to the acquisition of specific anti-VZV T cells, which are essential to prevent dissemination, ensure the resolution of acute infection and prevent reactivation. Among all PID described to date (13), susceptibility to VZV infection is heterogeneous (14). SCID and CID confer a high level of susceptibility to VZV as part of a broad predisposition to infection, further highlighting the major role of cellular immunity against VZV. CID constitute a large group of diseases, including some associated with a higher risk of extensive or disseminated varicella infection, such as autosomal recessive (AR) DOCK8 deficiency (15) and other PIDs related to actin-cytoskeleton abnormalities (16) (due to T-cell homing defect), in diseases with NK cell deficiencies among broader cellular deficiencies (AD GATA2 deficiency or AR MCM4 or GINS1 deficiencies) (17–19), AR DOCK2 deficiency, a PID that affect both innate and adaptive immunities, in which disseminated and fulminant varicella has been reported (20–22). A new PID conferring a narrow susceptibility to VZV has recently been described in patients with AR *POLR3A* and *POLR3C* deficiencies. The patients present a defective IFN type I and III production upon VZV infection (23) and displayed disseminated VZV with CNS or lung involvement.

Glucocorticoids have very broad immunosuppressive functions affecting both innate and adaptive immunity (24), which may account for the rapid dissemination of VZV and the severity of the infection in the AID group during the initial viremic phase, before the occurrence of a skin rash. The abdominal pain may be due to VZV replication in the digestive system (visceral varicella), as described in previous studies (25, 26). In our series, no DV has been reported in patients on chemotherapy for solid cancers. We cannot exclude an underreporting of such cases. However, the immunosuppressive effect of chemotherapies used in these conditions is probably weaker than that of current chemotherapy treatments for lymphoma and leukemia, which include steroids at various stages (27, 28). Indeed, the use of corticosteroids for immunosuppression was identified as a major risk factor for DV (22, 29).

All the PID patients with DV in our series had defective or absent T-cell immunity (CID or SCID). Almost all presented with an extended skin rash as the first symptom, but dissemination and prolonged infection were the general rule in this group of patients. We can, thus, speculate that, despite the intact innate immune responses engaged during the first phase of viremia, defective adaptive immune responses account for the dissemination and the lack of resolution of the infection, which followed a prolonged course. DV is rare in SCID patients (six out of 101 SCID patients diagnosed during the study period in the immune-hematology unit of Necker), but is a severe event. Mortality and the risk of sequelae were high. Because we included only cases of disseminated varicella infection, it may have introduced a selection bias towards the patients with the most marked PID.

The prompt diagnosis of varicella infection is of the utmost importance in these populations of patients at high risk, but is particularly challenging in patients with AID, in whom the clinical presentation differs from that classically observed (29, 30). The standard treatment for DV in immunocompromised patients includes prompt intravenous acyclovir treatment, initiated as soon as possible (31). Early treatment may improve prognosis (32). The addition of interferon-alpha, early in infection, may also improve outcome by helping to control of the initial viremia.

The prevention of VZV infection in the population at risk, with underlying PID or AID, is of considerable importance. Vaccination has been widely implemented in many countries and seems to have reduced the incidence of complications in otherwise healthy patients, and also in immunocompromised patients, through herd immunity (33). Unfortunately, the varicella vaccine currently available is a live vaccine that can cause infections in patients with profound T-cell immunodeficiency, as previously reported (34–36). The current recommendation in France is to propose anti-VZV vaccination to siblings and relatives of immunocompromised patients, with preventive treatment in cases of contact. Unfortunately, these recommendations are not fully applied, and are only partly efficient. In particular, for patients with PID, varicella infection may precede or lead to the diagnosis of PID. Neonatal screening for SCID should prevent primary infection in such patients before HSCT or gene therapy (37).

In conclusion, we highlight here major differences in the clinical presentations and outcomes of DV in patients with AID and PID, suggesting differences in the pathophysiology of the disease in these two groups. Abdominal pain is a major symptom in patients with AID, a prompt blood VZV PCR should be performed in the population at risk and acyclovir treatment initiated until the infection is ruled out, especially if liver enzymes are elevated. In the PID group, the prognosis of DV was worse for patients with SCID than for those with CID. The high mortality in this group of patients may reflect uncontrolled infection but IRIS, occurring at the time of immune recovery, should not be overlooked. The prevention of VZV infection in this high-risk population is of the utmost importance.

## Data Availability Statement

The original contributions presented in the study are included in the article/supplementary material. Further inquiries can be directed to the corresponding author.

## Ethics Statement

The studies involving human participants were reviewed and approved by Comité Local d’Ethique pour la Recherche Clinique; reference: CLEA-2016-029, October 12, 2016. Written informed consent to participate in this study was provided by the participants' legal guardian/next of kin.

## Author Contributions

PB and AG collected the clinical data. LP and BN supervised the work. All authors contributed to the article and approved the submitted version.

## Conflict of Interest

The authors declare that the research was conducted in the absence of any commercial or financial relationships that could be construed as a potential conflict of interest.
